# Supplementary material for: TRIB3 Modulates PPARγ-Mediated Growth Inhibition by Interfering with the MLL Complex in Breast Cancer Cells
Source: Int J Mol Sci. 2022 Sep 11;23(18):10535. doi: 10.3390/ijms231810535 (PMC9503934; doi:10.3390/ijms231810535)

SUPPLEMENTARY INFORMATION

TRAIN Consortium members:

Surname	First Name	Institution
Bhutia	Kunzangla	Instituto de Investigacion Santaria, Hospital Clinico SanCarlos
Brouard	Sophie	University of Nantes
Barril	Xavier	University of Barcelona
Carracedo	Arkaitz	Asociación Centro de Investigación Cooperativa en Biociencias
Castillo Lluva	Sonia	Instituto de Investigacion Santaria, Hospital Clinico SanCarlos
Danger	Richard A.E.D	University of Nantes
Day	Jack	Instituto de Investigacion Santaria, Hospital Clinico SanCarlos
Deshmukh	Sumeet R	The University of Sheffield
Feseha	Yodit	University of Nantes
Francis	Sheila	The University of Sheffield
Grzesik	Dominika J	William Harvey Research Institute, Queen Mary University, London
Hernandez Quiles	Miguel	Universitair Medisch Centrum Utrecht
Kalkhoven	Eric	Universitair Medisch Centrum Utrecht
Kiss-Toth	Endre	The University of Sheffield
Linford	Adam J	Institute for Diabetes and Cancer (IDC), Helmholtz Zentrum München

Martinez Campesino	Laura	The University of Sheffield
Metherall	Louise A	William Harvey Research Institute, Queen Mary University, London
Morris	Imogen	Universitair Medisch Centrum Utrecht
Niespolo	Chiara	The University of Sheffield
Pellegata	Natalia S	Institute for Diabetes and Cancer (IDC), Helmholtz Zentrum München
Ruiz Cantos	Miriam	William Harvey Research Institute, Queen Mary University, London
Salamanca Viloria	Juan	University of Barcelona
Satam	Swapna S	Institute for Diabetes and Cancer (IDC), Helmholtz Zentrum München
Scheideler	Marcel J.C.	Institute for Diabetes and Cancer (IDC), Helmholtz Zentrum München
Shahrouzi	Parastoo	Asociación Centro de Investigación Cooperativa en Biociencias
Shologu	Ziyanda	Universidade da Beira Interior
Shoulders	Carol C	William Harvey Research Institute, Queen Mary University, London
Sudbery	Ian	The University of Sheffield
Socorro	Silvia	Universidade da Beira Interior

Velasco	Guillermo	Instituto de Investigacion Santaria, Hospital Clinico SanCarlos
Villacanas Perez	Oscar	MindtheByte Ltd
Wilson	Heather	The University of Sheffield

Supplementary Table S1: Primers used for RT-qPCR

Gene name	Primer sequence (5'- 3')
hTRIB3	Fw: CTGAGTATCTCAGGTCCCACGT<br>Rv: AGGTGGAGCATTTCTCGCGCTA
hPPARG1	Fw: ATGGCATTATGAGACATCCC<br>Rv: CATAATGCCATCAGGTTTGGG
36B4	Fw: GGGAAGGTGTAATCCGTCTCCACAG<br>Rv: CGAGCGCGAGCATGTGT

Supplementary Table S2: Primers used for ChIP-RT-qPCR.

Gene name	Primer sequence (5'- 3')
PPARG pair 1	Fw: GGATGAGAGCTGGGGAGAAG<br>Rv: TCAGCCCAAGACCCCTTCA
PPARG pair 2	Fw: CTGAGGCAGGGTCATGGTC<br>Rv: AAGACCCCTTCACCCGGTC
HSCB	Fw: CACTCCCCCTTCAGCTCAAA<br>Rv: TGGGTGTTTGGTGGTTCGAA
Neg	Fw: GAGCCAGGGTTTCTCTGATTC<br>Rv: CCTCAGTGATCAGCCCTAAATG

Supplementary Table S3: Gene enrichment analysis.

Gene set	Direction	p-Value
Cytoplasmatic ribosomal proteins	Down	$8.42e^{-16}$
Apical junction	Up	0.0044
Cell differentiation index	Up	0.001
Translation factors	Down	0.001
White fat cell differentiation	Up	0.001
Myc target genes	Down	$1.19\ e^{-7}$
Oxidative phosphorylation	Down	0.000027
Kras signaling	Up	0.0013
Fatty acid metabolism	Down	0.066

Supplementary figure S1

A

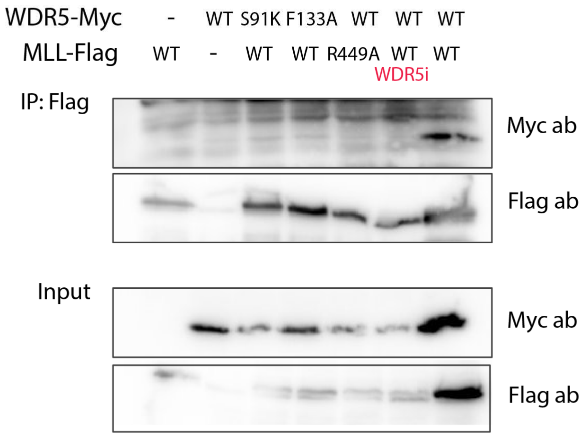

Supplement: Supplementary file 1 [file ijms-23-10535-s001.zip › ijms-1888939-supplementary.pdf]
